# Prognostic Utility of Albumin and the C-Reactive Protein-to-Albumin Ratio for Predicting Intravenous Steroid Response in Acute Severe Ulcerative Colitis

**DOI:** 10.3390/jcm15072730

**Published:** 2026-04-04

**Authors:** Sayar Süleyman, Pala Emin, Aykut Hüseyin, Ak Çağatay, Ölmez Şehmus, Özdil Kamil

**Affiliations:** 1 Department of Gastroenterology, VM Medical Park Pendik Hospital, 34899 İstanbul, Türkiye; cagatayak88@gmail.com; 2 Department of Gastroenterology, Sancaktepe İlhan Varank Training and Research Hospital, 34785 İstanbul, Türkiye; eminilepala@gmail.com; 3 Department of Gastroenterology, Ümraniye Training and Research Hospital, 34764 İstanbul, Türkiye; huseyin.ayk@gmail.com; 4 Department of Gastroenterology, University of Health Sciences, Adana City Training and Research Hospital, 01240 Adana, Türkiye; drsehmusolmez@gmail.com; 5 Department of Gastroenterology, Memorial Ataşehir Hospital, 34758 İstanbul, Türkiye; kamilozdil@gmail.com

**Keywords:** acute severe ulcerative colitis, intravenous steroid therapy, CRP-to-albumin ratio, albumin, steroid response, infliximab, prognostic biomarkers

## Abstract

**Background/Objectives:** Acute severe ulcerative colitis (ASUC) is a high-risk condition associated with intense inflammation and a substantial risk of early colectomy. Intravenous steroids (IVS) remain the first-line therapy; however, steroid nonresponse is common. While the C-reactive protein-to-albumin ratio (CAR) has been proposed as a prognostic marker, data on its dynamic changes and late-phase performance remain limited. This study aimed to evaluate the serial performance of albumin and CAR at days 3 and 7 and to assess their association with early and delayed steroid response in a real-world cohort of biologic-naive ASUC patients. **Methods:** Biologic-naive patients hospitalized with ASUC between 2010 and 2023 were retrospectively analyzed. Disease severity and treatment response were defined using Truelove–Witts and Oxford criteria. Clinical and biochemical parameters, including albumin, CAR, and neutrophil-to-lymphocyte ratio (NLR), were assessed at baseline and on days 3 and 7. Associations with steroid response were evaluated using ROC analysis and multivariable logistic regression. **Results:** Ninety-eight patients were included. The IVS response rate was 11.2% on day 3 and 56.1% by day 7. Among nonresponders, 25% required infliximab rescue therapy, and colectomy occurred in 12.2%. Although day 3 CAR showed a trend toward discrimination, this did not reach statistical significance (*p* = 0.098) and should be considered exploratory. By day 7, responders had significantly higher albumin levels and lower CAR values (*p* < 0.05). Albumin (AUC = 0.702) and CAR (AUC = 0.713) demonstrated comparable performance. In multivariable analyses, day 7 albumin and CAR were significantly associated with steroid response, whereas NLR was not associated. **Conclusions:** Albumin and CAR are clinically relevant biomarkers associated with steroid response in ASUC. Early changes, particularly in CAR, may provide preliminary signals of nonresponse but require cautious interpretation. In contrast, day 7 measurements appear to reflect ongoing inflammatory dynamics and treatment evolution rather than true baseline predictors. Serial assessment of these biomarkers may support treatment monitoring and help optimize the timing of rescue therapy.

## 1. Introduction and Aim

Ulcerative colitis (UC) is an immune-mediated inflammatory bowel disease that predominantly targets the colonic mucosa. Disease activity is determined through an integrated assessment of clinical symptom severity, systemic inflammatory findings, laboratory parameters, biochemical markers, and endoscopic activity. During the natural course of the disease, at least 25% of patients experience an acute severe ulcerative colitis (ASUC) episode requiring hospitalization [1].

ASUC is a serious clinical condition requiring urgent intervention; when not managed appropriately, the increased inflammatory burden may lead to colonic complications such as toxic megacolon, perforation, and massive bleeding, as well as systemic complications, including thromboembolic events. Despite the increasing availability of anti-cytokine therapies and biologic agents that modulate leukocyte migration, intravenous corticosteroid (IVS) therapy remains the cornerstone of first-line treatment. Therefore, the evaluation of therapeutic responses to IVS using objective criteria and timely transition to medical or surgical rescue therapies in nonresponsive cases is of critical clinical importance [2].

Although IVS remains the first-line treatment in ASUC, approximately 30–40% of patients do not respond and require rescue therapies such as infliximab or cyclosporine [3]. In a subset of these patients, surgical intervention becomes unavoidable. Accordingly, the early prediction of treatment responses and accurate estimation of the need for colectomy represent key steps in ASUC management.

Numerous studies have evaluated the use of clinical parameters (stool frequency and fever) and laboratory indicators (C-reactive protein, albumin, neutrophil and lymphocyte counts), the C-reactive protein-to-albumin ratio (CAR), and the neutrophil-to-lymphocyte ratio (NLR) to predict IVS responses in ASUC. However, real-world evidence pertaining to the predictive value of these biomarkers remains limited. Furthermore, data regarding the natural history of ASUC, early treatment response rates, and the prognostic performance of biochemical markers in Türkiye are particularly scarce [4].

Although CAR has been previously investigated as a prognostic marker in ASUC, most studies have relied on single time-point measurements and focused primarily on early (day 3) prediction [4]. Data on the dynamic changes in inflammatory biomarkers over time, particularly at later stages of treatment, remain limited, and real-world evidence in biologic-naive populations is scarce. Therefore, this study aimed to evaluate the serial prognostic performance of albumin and CAR on days 3 and 7 in biologic-naive ASUC patients, to determine early and late IVS response rates, and to assess their utility in identifying both early and delayed steroid responsiveness. In addition, the study explored the comparative predictive value of the NLR as a supplementary inflammatory marker.

## 2. Materials and Methods

### 2.1. Study Design and Patient Recruitment

In this study, patients who were diagnosed with UC between 2010 and 2023 based on clinical, laboratory, endoscopic, and histopathological findings, and who were hospitalized for the first time with ASUC according to the Truelove–Witts criteria, without any prior exposure to biologic therapy (biologic-naive), were retrospectively evaluated [5].

Patients meeting any of the following criteria were excluded: age < 18 years, failure to meet the ASUC diagnostic criteria, the absence of IV steroid therapy during hospitalization, pregnancy, active systemic infection (e.g., sepsis, tuberculosis) or infectious colitis (e.g., active CMV or Clostridium difficile infection), hematologic or other malignancies, advanced organ failure (cardiac, hepatic, renal), the presence of indeterminate colitis or Crohn’s disease, prior biologic therapy use, or incomplete clinical/laboratory data.

### 2.2. Definitions of Disease Severity and Treatment Response

The definition of ASUC was based on the presence of ≥6 bloody stools per day accompanied by at least one of the following systemic toxicity findings: body temperature > 37.8 °C, heart rate > 90 bpm, hemoglobin < 10.5 g/dL, erythrocyte sedimentation rate (ESR) > 30 mm/h, or C-reactive protein (CRP) > 30 mg/L [1].

The treatment response was assessed according to the Oxford criteria. On day 3, the presence of ≥8 stools per day or ≥3 stools per day accompanied by a CRP > 45 mg/L was considered “nonresponse”, while patients who did not meet these thresholds were classified as “responders” [6]. All patients underwent a complete day 3 assessment without exclusion, and no patient was transitioned to rescue therapy before this time point. Patients classified as nonresponders on day 3—but for whom rescue therapy was not initiated and IV steroid treatment was continued—were reassessed on day 7 using the same criteria.

The IVS response was assessed on days 3 and 7; the CAR and NLR were calculated on the same days [7,8].

Patients with missing day 3 or day 7 clinical/laboratory data were excluded from the corresponding analyses; no imputations were performed for missing values.

### 2.3. Data Collection and Clinical Assessments

All pre- and post-hospitalization patient data were reviewed retrospectively using inflammatory bowel disease outpatient clinic files, electronic medical records, and hospitalization reports. Demographic characteristics, disease duration, and pre-admission treatments were recorded. At the time of hospitalization, clinical parameters such as daily bloody stool frequency, fever, and other vital signs, as well as laboratory values including CRP, albumin, and complete blood count (CBC), were systematically documented.

During hospitalization, maintenance therapies other than oral corticosteroids and mesalamine, particularly azathioprine, were continued according to routine clinical practice.

### 2.4. Laboratory Measurements and Biomarker Calculations

C-reactive protein (CRP), albumin, and complete blood count (CBC) parameters were measured at admission and on the 3rd and 7th days of IVS therapy. CRP values were recorded in mg/L, and albumin values in g/dL.

The CRP-to-albumin ratio (CAR) was calculated by dividing the CRP (mg/L) value by the albumin (g/dL) value. The NLR was obtained by dividing the absolute neutrophil count by the absolute lymphocyte count [9]. All measurements were performed from the same blood sample to ensure analytical consistency.

Serum CRP and albumin levels were measured using standardized automated biochemical analyzers routinely employed in the hospital laboratory, and CBC parameters were obtained using automated hematology analyzers.

### 2.5. Outcomes and Follow-Up

In steroid-refractory patients, the presence of infectious colitis, administration of rescue therapies (infliximab, cyclosporine), and responses to these therapies were monitored.

The post-discharge follow-up duration was determined using outpatient clinic records and the date of the most recent hospital visit documented in the hospital information system.

### 2.6. Ethical Considerations

This study was conducted under the approval of the local ethics committee dated 11 January 2024 (Decision No: E.54132726000-233242701), and all procedures were carried out in accordance with the ethical principles and standards of the Declaration of Helsinki.

## 3. Statistical Analysis

Statistical analyses were conducted using IBM SPSS Statistics version 25.0 (IBM Corp., Armonk, NY, USA). The distributional characteristics of continuous variables were assessed using the Kolmogorov–Smirnov test. Variables that followed a normal distribution are reported as mean ± standard deviation, whereas non-normally distributed variables are expressed as median (minimum–maximum).

Between-group comparisons were performed using Student’s *t*-test or the Mann–Whitney U test for continuous variables, and the Chi-square test or Fisher’s exact test for categorical variables.

To identify independent determinants of response to IVS therapy, multivariable logistic regression analyses were performed separately for day 3 and day 7 based on the Oxford criteria.

Given the relatively limited number of events, particularly for day-3 responders, the number of variables included in the multivariable models was deliberately restricted to reduce the risk of overfitting. Variables were selected based on both clinical relevance and univariable screening (*p* < 0.10).

To further minimize collinearity and model overparameterization, two separate models were constructed: Model A (biochemical model), including CRP, albumin, neutrophil count, and lymphocyte count; and Model B (composite inflammation index model), including CAR and NLR.

Given the particularly low number of events on day 3, analyses at this time point were considered exploratory and interpreted with caution. For all regression analyses, odds ratios (ORs) and 95% confidence intervals (CIs) were reported. Model performance and fit were evaluated using the Akaike Information Criterion (AIC) and Nagelkerke’s pseudo-R^2^. This approach was intended to balance model parsimony with clinical interpretability while reducing the risk of overfitting.

The discriminatory performance of continuous biomarkers (CAR and NLR) in predicting the response to IVS therapy was assessed using receiver operating characteristic (ROC) curve analysis. For each ROC analysis, the area under the curve (AUC), optimal cut-off value (Youden index), sensitivity, specificity, and accuracy rates were calculated. When necessary, DeLong’s test was applied to statistically compare the ROC curves of different biomarkers. Statistical significance was set at a two-sided *p* < 0.05.

## 4. Results

**Figure 1:**
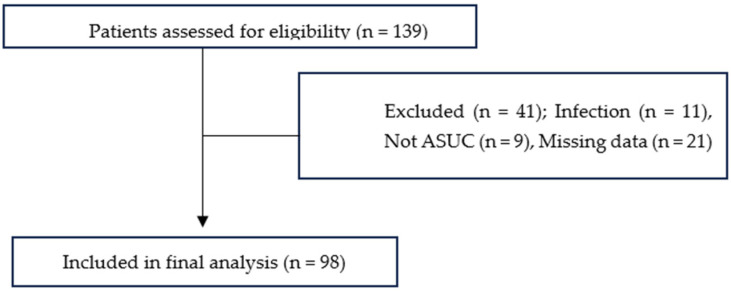
Flow diagram of patient selection. ASUC: Acute severe ulcerative colitis.

A total of 98 patients were included in the study (Figure 1). Of these, 62.2% were male (n = 61). Regarding the pattern of disease involvement, the majority of patients exhibited left-sided colitis (n = 72; 73.4%). The mean follow-up duration was 62.9 months (median: 60 months; range: 2–168 months), and the demographic and clinical characteristics of the cohort are summarized in Table 1.

The rate of response to IVS therapy on day 3 was 11.2% (n = 11). When evaluated on day 7, the cumulative response rate increased to 56.1% (n = 55). Among patients who remained unresponsive to IVS within the first 7 days (43.9%; n = 44), 25% (n = 11) received intravenous infliximab as rescue therapy, while others were managed with continued medical therapy and showed delayed clinical response. No patients received cyclosporine as rescue therapy. During follow-up, colectomy was performed in 12.2% (n = 12) of the entire cohort.

The mean time to clinical response was 11.8 days, indicating that a considerable proportion of patients achieved improvement beyond the first week of IVS therapy. The mean duration of IVS administration was 11.3 days. A total of 35 patients (35.7%) exhibited a late response to IVS, with the majority of delayed responders improving between days 10 and 14.

When patients who responded to IVS within the first 7 days were compared with those who did not, no significant differences in demographic or basic clinical characteristics were observed. However, albumin levels were significantly higher in responders (*p* = 0.016).

Although CAR values were lower in responders on day 3 (mean, 0.26 ± 0.19; median, 0.17), they were markedly elevated in nonresponders (1.25 ± 1.46; median, 0.63). However, this difference did not reach statistical significance (*p* = 0.098).

A significant difference in CAR values was detected on day 7; CAR levels in nonresponders (1.29 ± 1.69; median 0.63) were substantially higher than those in responders (0.40 ± 0.42; median, 0.21) (*p* < 0.05). These findings are presented in Table 2.

The NLR values on both days 3 and 7 did not differ significantly between responders and nonresponders (*p* > 0.05).

**Figure 2:**
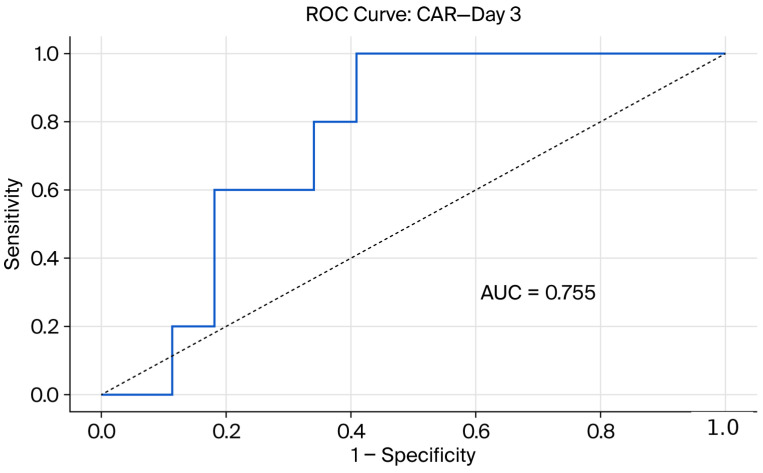
Receiver operating characteristic (ROC) curve demonstrating the performance of the C-reactive protein-to-albumin ratio (CAR) in predicting IVS response on day 3.

**Figure 3:**
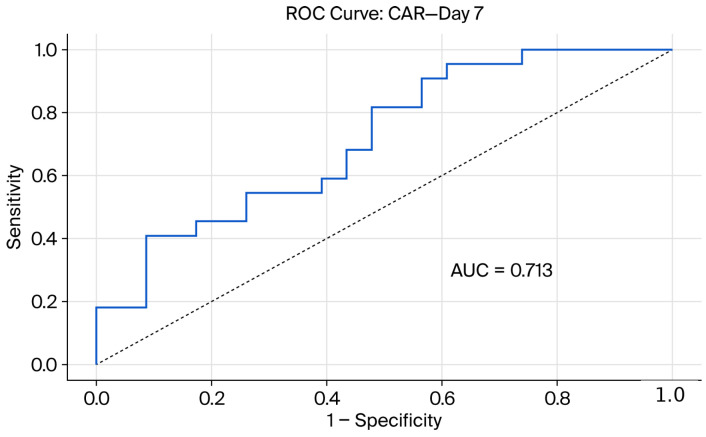
Receiver operating characteristic (ROC) curve demonstrating the performance of the C-reactive protein-to-albumin ratio (CAR) in predicting IVS response on day 7.

**Figure 4:**
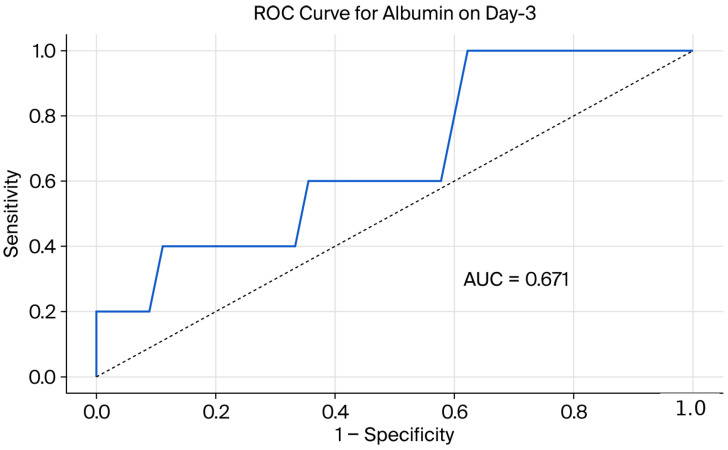
Receiver operating characteristic (ROC) curve demonstrating the performance of serum albumin in predicting IVS response on day 3.

**Figure 5:**
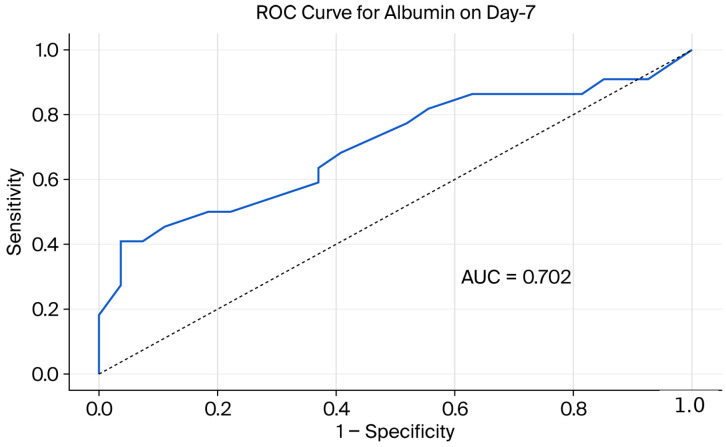
Receiver operating characteristic (ROC) curve demonstrating the performance of serum albumin in predicting IVS response on day 7.

To evaluate the biochemical predictability of the steroid response, ROC analyses were performed for albumin and CAR values. The diagnostic performance of these markers in predicting the steroid response on days 3 and 7 is presented in Table 3 and Table 4 and Figure 2, Figure 3, Figure 4 and Figure 5. ROC analysis for day 3 CAR was also performed; however, the results should be interpreted in the context of the non-significant group comparison.

Among the 11 patients who received infliximab rescue therapy, 6 (54.5%) achieved clinical response. In exploratory subgroup analyses, neither CAR nor NLR differed significantly between infliximab responders and nonresponders at day 3 or day 7 (all *p* > 0.05).

In ROC curve comparisons conducted using DeLong’s test on day 7, no significant difference between the predictive performances of albumin (AUC = 0.702) and CAR (AUC = 0.713) was observed (*p* > 0.05).

Multivariable logistic regression analyses yielded the following results (Table 5 and Table 6). Model A (CRP, albumin, neutrophils, and lymphocytes): On day 3, none of the parameters emerged as independent predictors. On day 7, albumin level was identified as a significant positive predictor (OR = 10.92; 95% CI: 1.57–76.24; *p* = 0.016). Model B (CAR, NLR): On day 3, CAR demonstrated a negative trend but did not reach statistical significance (OR = 0.07; *p* = 0.158). On day 7, CAR was found to be a significant independent negative predictor (OR = 0.29; 95% CI: 0.09–0.94; *p* = 0.040). The NLR was not significant at either time point.

## 5. Discussion

Intravenous corticosteroid therapy remains the cornerstone of treatment in ASUC. Current international guidelines recommend evaluating treatment responses early, particularly on day 3, using the Oxford criteria [10,11,12]. This approach aims to facilitate the rapid identification of early nonresponse and to enable a timely transition to rescue therapy without delay.

In the present study, the steroid response rate on day 3—based on the Oxford criteria—was found to be 11%. Although this value falls below the 25–40% range reported in the literature [10,13], the relatively low day 3 response rate observed in this study may be explained by several factors. All patients were evaluated at day 3 without early exclusion or transition to rescue therapy, ensuring a complete assessment of treatment response. The strict application of the Oxford criteria may have resulted in a more conservative classification of early response. In addition, prior corticosteroid use before hospitalization may have influenced baseline inflammatory status and early response dynamics. This may also explain the relatively higher proportion of delayed responders observed in our cohort.

The increase in response rate to 56.1% by day 7 suggests that a subset of patients classified as “partial responders” on day 3 experienced marked clinical or biochemical improvement between days 5 and 7. The absence of real-world evidence on early IVS response rates in Turkish ASUC patients further underscores the significance of this study.

Among the principal biochemical markers reflecting the degree of systemic inflammation in UC are CRP and albumin levels. CRP is synthesized in hepatocytes via an IL-6-mediated acute phase response and is closely associated with disease activity [14,15]. Elevated CRP levels are widely considered an important indicator of active mucosal inflammation, steroid refractoriness, and the likelihood of early surgical intervention [6,16]. However, because of interindividual variability and its kinetic properties, CRP alone has recognized limitations in clinical decision making [17].

Serum albumin is a negative acute-phase reactant and an important indicator of systemic inflammation, catabolic stress, and intestinal protein loss. Hypoalbuminemia may arise from mechanisms such as increased intestinal permeability, protein-losing enteropathy, and malnutrition; it is strongly associated with disease activity, treatment response, and colectomy risk [18,19,20].

The CAR, by combining both inflammatory burden and systemic response into a single index, has gained increasing importance in predicting prognosis and treatment response in ulcerative colitis [21]. CAR has been shown to possess prognostic value not only in UC but also in sepsis [22], acute pancreatitis [23], and solid organ malignancies [24], supporting its ability to sensitively reflect systemic inflammation.

In this study, CAR showed a trend toward discrimination in early (day 3) treatment responses; however, this finding did not reach statistical significance and should be considered exploratory. In contrast, the observation that higher albumin levels and lower CAR values on day 7 were associated with steroid responsiveness reflects a more robust and clinically meaningful pattern. This trend aligns with prior studies reporting associations between hypoalbuminemia, elevated CAR levels, and adverse clinical outcomes [19].

The upward trend in albumin levels by day 7 may reflect the initiation of mucosal healing as systemic inflammation subsides. Recent prospective data have demonstrated a strong association between serum albumin levels and mucosal healing in ulcerative colitis, particularly in earlier disease stages [18]. Gibson et al. [21] further emphasized that, although decreases in CRP may reflect early clinical improvement, increases in albumin better represent the “sustainability of biological recovery”.

An important consideration in interpreting our findings is the distinction between predictive and monitoring biomarkers. While day 3 parameters may have a role in early prediction of steroid nonresponse, biomarkers measured at day 7 are more likely to reflect the ongoing inflammatory trajectory and treatment response rather than serve as true baseline predictors. Therefore, albumin and CAR at day 7 should be interpreted primarily as dynamic indicators of disease evolution and therapeutic effect, rather than independent predictive factors in the strict sense. This distinction is clinically relevant, as it supports the use of these markers in treatment monitoring and decision refinement, rather than in initial risk stratification alone.

The study period (2010–2023) spans a time during which substantial shifts occurred in ASUC treatment algorithms. Earlier European Crohn’s and Colitis Organisation (ECCO) and British Society of Gastroenterology (BSG) guidelines recommended response assessment within a flexible 3–7-day interval [11,12]. However, more recent guidelines—ECCO 2022 and BSG 2025—advise initiating infliximab or cyclosporine without delay on day 3 in nonresponders, while the American College of Gastroenterology recommends evaluation at day 5 [25,26,27].

The observation that a subset of patients categorized as “partial responders” on day 3 showed marked improvement between days 5 and 7 is consistent with clinical practice patterns of that period and reflects real-world disease behavior [13]. This finding further highlights the importance of dynamic biomarker evaluation.

Dynamic assessment of treatment responses in ASUC—particularly through biochemical indicators such as CRP, albumin, and CAR—is of critical importance. In cases where clinical response is not evident by day 3, short-term changes in these markers may reflect the underlying biological activity of the disease and contribute to personalized treatment strategies. The Oxford criteria, developed by Travis and colleagues, demonstrated that the day 3 clinical response is a strong predictor of colectomy risk. Turner et al. similarly emphasized the predictive value of day 3 CRP level in determining the need for rescue therapy [6,10].

Approximately one-third of patients do not respond to IVS. In this group, rescue therapies such as intravenous cyclosporine or infliximab achieve disease control in approximately 60% of cases, while colectomy becomes unavoidable in 10–15% [28,29,30]. In our study, the absence of an association between demographic factors and steroid response aligns with similar findings reported by Ho et al. [19].

Although the NLR has been proposed as a potential predictor of steroid response in some studies [31,32], no significant difference in NLR values at either time point in this study was observed. Several factors may explain this finding. First, NLR is influenced by multiple systemic and treatment-related variables, including corticosteroid-induced neutrophilia and lymphopenia, which may reduce its specificity as a marker of disease activity in the acute setting. In addition, a considerable proportion of patients in our cohort had received corticosteroids and/or azathioprine prior to hospitalization, which may have further altered baseline leukocyte dynamics and attenuated the discriminatory capacity of NLR. This may also represent a potential confounding factor influencing the interpretation of inflammatory biomarkers in this cohort. Second, compared with composite markers such as CAR, NLR may less accurately reflect the combined effects of inflammatory burden and nutritional status. Third, variability in cut-off values and the relatively small sample size may have limited the ability to detect a significant association. These findings suggest that NLR may have limited utility as a standalone marker in this clinical context.

In our study, approximately one-quarter of patients required infliximab as rescue therapy, with a response rate of 54.5%, consistent with previous reports [28,32,33,34]. However, this finding should be interpreted cautiously given the small sample size (n = 11), which limits the reliability of this estimate. In addition, CAR and NLR were not significantly associated with infliximab response in this subgroup, and these findings should be considered exploratory.

Prospective trials have demonstrated that infliximab reduces colectomy rates in steroid-refractory ASUC, supporting this finding. A colectomy rate of 12.2% observed in our study is in line with rates reported in selected real-world ASUC cohorts, where colectomy rates of approximately 15–20% have been described [1,35].

Because ‘late steroid response’ is not consistently defined across studies, direct comparison of response rates is limited. The delayed improvement observed in our cohort likely reflects real-world practice patterns during the study period and the frequent use of concomitant azathioprine, which may be associated with a slower trajectory of inflammatory control.

This study has several limitations. First, its retrospective design may have increased the risk of measurement error and selection bias due to reliance on the accuracy of clinical and biochemical records. The exclusion of patients with missing day 3 and/or day 7 follow-up data may have introduced additional selection bias. Second, although the use of single-center data may have provided a more homogeneous patient population, it limits the generalizability of the findings. Third, the relatively small number of events, particularly on day 3, may increase the risk of overfitting. This may also be reflected in the relatively wide confidence intervals observed in some regression analyses. Despite efforts to limit model complexity, day 3 findings should be interpreted cautiously. The absence of significant predictors and the ROC performance at this time point likely reflect limited statistical power and should be considered exploratory. In contrast, day 7 results appear more consistent. Finally, given the long study period (2010–2023), substantial changes in ASUC management guidelines and clinical practices may have contributed to cohort heterogeneity and temporal variation in biomarker behavior and treatment responses. A formal temporal subgroup analysis was not performed due to the limited sample size, which may reduce the reliability of such comparisons. Nevertheless, despite these limitations, the study provides valuable real-world evidence regarding ASUC management in Türkiye.

## 6. Conclusions

In conclusion, this study found a 56% steroid response rate within the first 7 days in patients with ASUC. Albumin and CAR emerged as clinically meaningful biomarkers associated with steroid responsiveness. Although a higher day 3 CAR value (≥0.56) showed high specificity for early nonresponse, this finding did not reach statistical significance and should therefore be considered exploratory and hypothesis-generating. In contrast, day 7 albumin ≤ 3.5 g/dL and CAR ≥ 1.4 appear to reflect persistent inflammatory burden and ongoing treatment dynamics. These findings suggest that, while early changes may provide preliminary signals, biomarkers measured at later time points are more indicative of treatment evolution rather than true baseline predictors. Therefore, serial assessment of albumin and CAR may provide clinically valuable insight for treatment monitoring and for optimizing the timing of transition to rescue therapy.

## Figures and Tables

**Table 1:** Demographic and baseline characteristics of the patients.

	Patients (n = 98)
Age of patients at admission (years)	40 ± 13.9 (mean)
Gender n (%)	
Male	61 (62.2%)
Female	37 (37.8%)
Age at diagnosis (years)	36.6 ± 13.2 (mean); 33.0 [27.0–44.0] (median)
Family history of inflammatory bowel disease (IBD) n (%)	8 (8.2%)
Extent of disease (Montreal Classification) n (%)	Left-sided colitis: 72 (73.4%) Extensive colitis: 26 (26.6%)
Pre-admission disease duration (months)	52.2 ± 77.4 (mean); 24.0 [12.0–72.0] (median)
Pre-admission treatment: n (%)	
Without treatment	11 (11.2%)
Mesalamine	16 (16.3%)
Oral steroid use	16 (16.3%) *
Azathioprine	71 (72.5%) *
Smoking history n (%)	19 (19.4%)
Post-hospital follow-up duration (months)	62.9 ± 41.5 (mean); 60.0 [24.0–94.5] (median)

* Treatment categories are not mutually exclusive; patients may have received combination therapy (e.g., mesalamine with corticosteroids and/or azathioprine).

**Table 2:** Analysis of laboratory parameters according to treatment response on day 7.

Time	Parameters	IVS Responder Mean ± SD (Median)	IVS Nonresponder Mean ± SD (Median)	*p*-Value
Admission	CRP (mg/L)	29.4 ± 24.5 (21.0)	58.9 ± 59.6 (41.0)	0.217
	Albumin (g/dL)	3.36 ± 0.60 (3.30)	3.11 ± 0.65 (3.10)	0.290
	CAR	0.847 ± 0.701 (0.581)	2.06 ± 2.29 (1.22)	0.179
	NLR	5.11 ± 3.41 (5.16)	4.40 ± 2.82 (3.90)	0.462
Day 3	CRP (mg/L)	13.1 ± 8.3 (12.5)	33.6 ± 34.4 (18.0)	0.127
	Albumin (g/dL)	3.32 ± 0.57 (3.15)	3.07 ± 0.62 (3.10)	0.457
	CAR	0.316 ± 0.177 (0.277)	1.31 ± 1.47 (0.677)	0.098
	NLR	3.69 ± 2.11 (3.45)	3.98 ± 2.65 (3.21)	0.966
Day 7	CRP (mg/L)	10.1 ± 11.3 (5.0)	29.0 ± 37.4 (9.15)	**0.004**
	Albumin (g/dL)	3.15 ± 0.56 (3.15)	2.78 ± 0.39 (2.80)	**0.016**
	CAR	0.396 ± 0.420 (0.214)	1.29 ± 1.69 (0.633)	**0.015**
	NLR	4.25 ± 3.29 (3.11)	4.09 ± 2.00 (3.82)	0.284

CRP: C-reactive protein; CAR: C-reactive protein-to-albumin ratio; IVS: intravenous corticosteroid therapy; NLR: neutrophil-to-lymphocyte ratio. *p*-values shown in bold denote statistically significant results (*p* < 0.05).

**Table 3:** Diagnostic performance of biomarkers in predicting day 3 response to IVS.

Parameters	*p*-Value	Cut-Off	AUC	Sensitivity (%)	Specificity (%)	Accuracy (%)
CRP (mg/L)	0.127	>45	-	-	-	-
Albumin (g/dL)	0.457	≥2.9	0.671	100	37.8	44
CAR	0.098	≥0.56	0.755	59	100	63
NLR	0.966	-	-	-	-	-

CRP: C-reactive protein; CAR: C-reactive protein-to-albumin ratio; NLR: neutrophil-to-lymphocyte ratio; IVS: intravenous corticosteroid therapy.

**Table 4:** Diagnostic performance of biomarkers in predicting day 7 response to IVS.

Parameters	*p*-Value	Cut-Off	AUC	Sensitivity (%)	Specificity (%)	Accuracy (%)
CRP (mg/L)	**0.004**	>45	-	-	-	-
Albumin (g/dL)	**0.016**	≥3.5	0.702	40.9	96.3	71.4
CAR	**0.015**	≥1.40	0.713	39	95	67
NLR	0.284	-	-	-	-	-

CRP: C-reactive protein; CAR: C-reactive protein-to-albumin ratio; NLR: neutrophil-to-lymphocyte ratio; IVS: intravenous corticosteroid therapy. *p*-values shown in bold denote statistically significant results (*p* < 0.05).

**Table 5:** Model A—independent predictors of IVS response (multivariable logistic regression analysis).

Variable	OR	95% CI	*p*-Value	Time
CRP (mg/L)	1.08	0.75–1.53	0.71	**Day 3**
Albumin (g/dL)	0.93	0.69–1.24	0.65	
Neutrophil (×10^3^/µL)	0.87	0.65–1.16	0.33	
Lymphocyte (×10^3^/µL)	0.24	0.03–1.78	0.16	
CRP (mg/L)	0.97	0.72–1.30	0.83	**Day 7**
Albumin (g/dL)	10.92	1.57–76.24	**0.016**	
Neutrophil (×10^3^/µL)	1.10	0.89–1.36	0.38	
Lymphocyte (×10^3^/µL)	1.41	0.62–3.20	0.41	

CRP: C-reactive protein; IVS: intravenous corticosteroid therapy. *p*-values shown in bold denote statistically significant results (*p* < 0.05).

**Table 6:** Model B—independent predictors of intravenous steroid (IVS) response (multivariable logistic regression analysis).

Variable	OR	95% CI	*p*-Value	
CAR	0.07	0.00–8.10	0.16	**Day 3**
NLR	0.97	0.66–1.41	0.87	
CAR	0.29	0.09–0.94	**0.040**	**Day 7**
NLR	1.11	0.77–1.60	0.58	

CAR: C-reactive protein-to-albumin ratio; NLR: neutrophil-to-lymphocyte ratio; IVS: intravenous corticosteroid therapy. *p*-values shown in bold denote statistically significant results (*p* < 0.05).

## Data Availability

The data supporting the findings of this study are available from the corresponding author upon reasonable request.
